# Editorial to Special Issue Toxic Metals, Chronic Diseases and Related Cancers

**DOI:** 10.3390/toxics10030125

**Published:** 2022-03-05

**Authors:** Soisungwan Satarug

**Affiliations:** Kidney Disease Research Collaborative, Centre for Health Services Research, Translational Research Institute, Brisbane, QLD 4102, Australia; sj.satarug@yahoo.com.au

In this Special Issue, entitled “Toxic Metals, Chronic Diseases and Related Cancers”, there are 19 published manuscripts, including reports of environmental exposure monitoring [1,2] and food safety surveillance [3,4]; reviews focusing on health risks of chronic exposure to cadmium and lead [5], experimental studies on the toxicity of cadmium, lead or mercury in gonads [6], and evidence that cadmium exposure may be one of the environmental factors contributing to hyperglycemia, insulin resistance and diabetes [7]; a mathematical model of the oral glucose tolerance test [8]; epidemiological studies examining health impacts of cadmium, lead, and mercury [9,10,11,12,13,14,15]; and reports concerning the immunotoxicity of high exposure to lead [16], the effects of cadmium on protein reabsorption by kidney tubular epithelial cells [17], the cytotoxicity of hexavalent chromium [18], and the carcinogenicity of aluminum in vivo [19].

*Environmental Monitoring*

To assess the health impact of a hazardous waste incinerator in Constantí, Catalonia, Spain, Esplugas et al. quantified elemental composition (As, Be, Cd, Cr, Hg, Mn, Ni, Pb, Sn, Tl and V) of scalp hair samples from schoolchildren living in close proximity to the incinerator and compared data to those recorded for schoolchildren in other areas [1]. Arsenic, beryllium and thallium were not detected, but cadmium and vanadium were found in 39% and 24.5% of samples, respectively. In order of high to low, the elemental contents of samples analyzed were Pb > Hg > Ni > Sn > Mn > Cr. These elemental contents were similar or lower than those reported for scalp hair samples from other areas [1]. In the most recent autopsy series describing tissue metal content, García et al. report that cadmium was detected in 30%, 30%, 100%, and 100% of brain, bone, liver and kidney samples, while lead was detected in 50%, 95%, 80%, and 85% of brain, bone, liver and kidney samples, respectively [2]. Preferential accumulations of cadmium in kidneys and lead in bone were evident [2].

*Food Safety Surveillance and Mortality from Kidney Failure*

Horiguchi et al. report cadmium intake levels among women living in two areas of Japan with recognized cadmium pollution in excess of the current tolerable intake level of 0.83 µg/kg bw/d [3]. In one area where rice cadmium content ranged from below 0.02 to 0.971 mg/kg, the average cadmium intake was 55.7 µg/d (1.03 µg/kg body weight per day). In the other area, where rice cadmium content varied between 0.008 and 0.687 mg/kg/d, the average cadmium intake was 48.7 µg/d (0.86 µg/kg bw/d). The percentage of rice samples containing cadmium above the Codex safety standard of 0.4 mg/kg was 8.2% in one area and 5.8% in the other.

In another Japanese area polluted by cadmium, 12 of 22 villages produced rice with cadmium contents above the 0.4 mg/kg standard. In a 35-year follow-up study of 2602 residents in this area (1169 men and 1433 women), Nishijo et al. found that lifetime cadmium intake ≥ 1 g was associated with a 49% increase in mortality from kidney failure among women. They also found that urinary cadmium levels ≥ 10 µg/g creatinine at baseline (35 years earlier) were associated with a 33% increase in all-cause mortality in women. Of note, a lifetime cadmium intake of 1 g is half of a “tolerable” lifetime intake guideline of 2 g.

*Clinical Kidney Function (GFR) Deterioration*

A review by Satarug et al. summarizes dietary sources and urine- and blood-based exposure measures of cadmium and lead, the two most prevalent toxic metals in humans. The review also examines the health risks of chronic exposure to cadmium and lead and the pathogenesis of cadmium-induced GFR reduction [5]. An evolving body of evidence links these metals to chronic kidney disease and mortality from cancer and heart disease [5]. Chronic kindey disease is defined as a fall of GFR below 60 ml/min/1.73m^2^ or an increment of albumin-to-creatinine ratio to 30 mg/ g creatinine in women and to ≥ 20 mg/ g creatinine in men that persists for at least three months.

In a prospective cohort study of 601 Mexican children, Rodríguez-López et al. report means for cadmium intake of 4.4 µg/d at baseline and 8.1 µg/d after nine years [10]. The respective percentages of intake levels exceeding a tolerable level at baseline and at nine years of the cohort were 64% and 16%. A dietary transition to sweets, lettuce, and sandwiches as the main cadmium sources was seen at 4 years of age which coincided with a rise of an obesity prevalence from 18% to 46.8% at 9 years of age. Because a tolerable intake guideline was on a body weight basis, the % of intake levels exceeding a tolerable range fell while mean intake rose. Cadmium intake levels among children 8–12 years old showed a marginally inverse association with estimated glomerular filtration rate (eGFR). 

Satarug et al. report a significant effect on eGFR of low environmental exposure to cadmium and lead among 392 Thai subjects (mean age 34.9 years) [11]. The mean for eGFR in subjects with urinary cadmium in the fourth quartile was, respectively, 4.65 and 4.94 mL/min/1.73 m^2^ lower than those with urine cadmium in the first quartile (*p* = 0.021) and the second quartile (*p* = 0.011). In an in-depth analysis of data from 704 persons, of which 172, 310, and 222 were drawn, respectively, from the low, moderate, and high exposure areas of Thailand, a decrease in eGFR was the result of loss of intact nephrons due to extensive injury to kidney tubular cells caused by cadmium [12]. An elevation of β_2-_microglobulin (β_2_MG) excretion was speculatively due to effects of cadmium on both tubular reabsorption and nephron number.

Fujishiro et al. report that reabsorption of β_2_MG and metallothionein (MT) by immortalized cells derived from human proximal tubule dropped after incubation with cadmium for 3 days [17]. A similar result was seen when S1 and S2 cell culture models of mouse proximal tubule were tested. Renal reuptake of iron (bound to transferrin) occurring at proximal tubule did not seem to be affected by cadmium. However, reabsorption of albumin and transferrin by these cells was not affected by cadmium. A question remains with regard to the specificity of this cadmium effect. A recent study suggested that reabsorption of β_2_MG and MT may occur mainly at the distal tubule and collecting duct, where other receptor-mediated endocytosis systems are expressed [20].

*Reproductive Health, Low Infant Birthweight, and Abnormal Growth*

A review by Massányi et al. summarizes experimental studies showing the effects of cadmium, lead or mercury on the function of gonads, where female gametes (oocytes), male gametes (spermatozoa), and sex hormones are formed [6]. The reported effects of cadmium in ovaries include reduced follicular growth, follicular atresia, and prolonged estrus cycle. In testes, notable effects of cadmium are degeneration of the seminiferous tubules, disorganization of germinal epithelium in seminiferous tubules and abnormal spermatogenesis [6]. It is argued that low environmental exposure to cadmium, lead or mercury may contribute to the worldwide decline of human fertility which currently stands at 15% of childbearing age couples.

The Mediterranean diet is rich in iron and selenium, elements that in theory can reduce cadmium absorption. Gonzalez-Nahm et al. undertook a prospective cohort study of 185 mother–infant pairs of central North Carolina, USA to assess whether a Mediterranean diet during pregnancy modified the effect of prenatal cadmium exposure on birth outcomes [9]. The 25th, 50th, and 75th percentile levels of prenatal blood cadmium levels among cohort participants were 0.12, 0.24 and 0.46 µg/L, respectively. For the entire group, prenatal blood cadmium levels ≥ 0.46 µg/L were associated with low infant birthweight (≤2500 g). In a subgroup analysis, the effect size of cadmium in Mediterranean diet adherence and non-adherence groups was similar. In this study, maternal adherence to a Mediterranean diet pattern did not mitigate the effect of cadmium on infant birthweight. However, the effect size observed was large (β = −210; 95% CI: −332, −88; *p* = 0.008), and it warrants further research to find dietary patterns that can diminish the absorption of dietary cadmium during pregnancy. 

In a study of 311 children (151 girls and 160 boys), aged 3–7 years, from a coastal area of Thailand, Yimthiang et al. report a 2-fold increase in the risk of stunted growth among children who had high blood lead levels (≥5 µg/dL) [14]. Milk consumption reduced the risk of abnormal growth by 43%. It is likely that calcium in milk reduced lead absorption by competing with lead for the metal transporters responsible for the absorption of lead. Another protective mechanism of milk might be due to organic substances that chelate lead and reduce its absorption. The authors also link high blood lead in children to parental occupations, such as fishing net production, that involved the use of lead weights.

*Insulin Resistance and Diabetes*

A review by Buha et al. discusses the evidence that cadmium exposure may contribute to hyperglycemia, insulin resistance and diabetes [7]. The authors describe various obstacles in their effort to derive an exposure limit for the insulin resistance associated with chronic low environmental exposure to cadmium. The predicaments are analogous to research into the threshold levels for endocrine-disrupting chemicals. The authors argue that a hazard-based, no-threshold approach should be applied when glucose homeostasis and insulin resistance are considered [7].

To overcome the limitation of a conventional approach to studying a complex physiological process in which many organs and tissues are involved, Rocca et al. use mathematical modeling to interpret oral glucose tolerance test data [8]. With a model incorporating four ordinary differential equations, they show that perinatal exposure to low-level cadmium in mother’s milk reduced pancreatic β-cell sensitivity to glucose [8]. A review by Mari et al. presents an in-depth discussion on mathematical modeling to investigate complex physiological processes [21].

*Stroke, Hyperlipdemia, and Liver Injury*

In a case–control study of 92 stroke patients and 83 controls who were residents of the Canary Islands of Spain, Medina-Estévez et al. observed an association between high blood lead and an increase in risk of stroke by 65% when a univariate analysis was used [13]. In a multivariate analysis, high blood lead was associated with a 91% increase in risk of stroke after controlling for other stroke risk factors (smoking, hypertension, dyslipidemia and coronary cardiopathy). In contrast, high blood levels of gold and cerium were associated with a decrease in risk of stroke by 19% and 50%, respectively. The authors suggest that higher blood gold and cerium levels may explain the lower incidence of stroke in both men and women in the Canary Islands, compared to other regions of Spain. Further research is required, given that gold and cerium, like lead, are cumulative elemental chemicals, and little is known about their sources and effects in the body. 

Lee et al. assessed the potential health effect of mercury intake among 6454 participants in the Korean National Environmental Health Survey [14]. They found higher means for blood mercury in women than men (3.70 vs. 2.63 μg/L), and noted that high blood mercury was associated, respectively, with 10.5% and 34.5% increases in risk of hyperlipidemia and liver injury (elevated plasma levels of liver enzymes).

*Immunosuppression and Cancer*

Pukanha et al. assessed immunotoxicity of occupational exposure to high levels of lead using white blood cells samples from a group of boatyard workers (*n* = 14) and an age-matched control group of farmers (*n* = 16) [16]. The median blood lead concentration was 37.1 µg/dL in workers and 4.3 µg/dL in controls. Compared to controls, workers had 8.4% fewer active phagocytic cells and 33.9% fewer cytotoxic T (Tc) cells, but the percentage of regulatory T (Treg) cells was higher by a factor of 2.7. In all subjects, blood lead levels showed positive correlations with the percentages of Treg cells (*r* = 0.843, *p* < 0.001) and interleukin-4 (*r* = 0.473, *p* = 0.041) while showing an inverse correlation with the percentages of Tc cells (*r* = −0.563, *p* = 0.015). Thus, chronic high exposure to lead may suppress cellular immunity while causing a shift towards humoral immunity. The immunosuppressive conditions accompanying lead exposure may increase the risks of infection and cancer.

Ma et al. employed classic molecular techniques to identify a specific molecular entity responsible for the cytotoxicity of hexavalent chromium in human liver cell line L02 [18]. It was shown that mitochondrial fission and fragmentation seen in L02 hepatocytes treated with chromium was mediated by Dynamin-Related Protein 1 (DRP1). A translocation of DRP1 from the cytoplasm to mitochondrial inner membrane occurred following excessive production of reactive oxygen species induced by chromium treatment.

García-Alegría et al. evaluated the effects of aluminum (as AlCl_3_) alone or in com-bination with N-nitroso-N-methylurea (NMU), a chemical used in experimental induction of breast cancer [19]. Aluminum was administered to Sprague Dawley rats by gavage 5 days/week for 90 days, at a dose of 10 mg/day/kg of body weight, while NMU was administered via intraperitoneal route 50 and 70 days of age, at a dose of 50 mg/kg of body weight. Unexpectedly, aluminum accumulation was higher in mammary gland tissue from the group treated with aluminum only (38.2 vs. 12.3 µg/g). Based on an analysis of transcript levels, the involvement of SCL11A2 (divalent metal transporter 1) in the uptake of aluminum in mammary gland tissue was unlikely. Aluminum treatment alone induced minimum-to-moderate intraductal cell proliferation, lymph node hyperplasia, and serous gland adenoma.

Environmental exposure is estimated to account for 70–90% of the risks of acquiring chronic ailments. Presently, chronic kidney disease affects 8% to 16% of the world population, while the global prevalence of infertility among childbearing age couples is around 15%. Collectively, data presented in this Special Issue indicate that environmental exposure to toxic metals may contribute to these looming statistics. Alarming evidence suggests that exposure to cadmium may affect every stage of life, and that exposure in early life may determine susceptibility to certain diseases in adulthood. Prevention of these outcomes requires avoidance of further environmental contamination, minimization of exposure, and reduction of toxic metals in food crops to the lowest achievable levels. 
